# Acute and Chronic Effects of Endurance Running on Inflammatory Markers: A Systematic Review

**DOI:** 10.3389/fphys.2017.00779

**Published:** 2017-10-17

**Authors:** Edilberto S. Barros, Dahan C. Nascimento, Jonato Prestes, Otávio T. Nóbrega, Claúdio Córdova, Fernando Sousa, Daniel A. Boullosa

**Affiliations:** ^1^Physical Education, Catholic University of Brasilia, Brasília, Brazil; ^2^Medical Sciences, University of Brasilia, Brasília, Brazil; ^3^Sport and Exercise Science, College of Healthcare Sciences, James Cook University, Townsville, QLD, Australia

**Keywords:** running, inflammation, marathon, half-marathon, athletes, immunology

## Abstract

In order to understand the effect of endurance running on inflammation, it is necessary to quantify the extent to which acute and chronic running affects inflammatory mediators. The aim of this study was to summarize the literature on the effects of endurance running on inflammation mediators. Electronic searches were conducted on PubMED and Science Direct with no limits of date and language of publication. Randomized controlled trials (RCTs) and non-randomized controlled trials (NRCTs) investigating the acute and chronic effects of running on inflammation markers in runners were reviewed by two researchers for eligibility. The modified Downs and Black checklist for the assesssments of the methodological quality of studies was subsequently used. Fifty-one studies were finally included. There were no studies with elite athletes. Only two studies were chronic interventions. Results revealed that acute and chronic endurance running may affect anti- and pro-inflammatory markers but methodological differences between studies do not allow comparisons or generalization of the results. The information provided in this systematic review would help practitioners for better designing further studies while providing reference values for a better understanding of inflammatory responses after different running events. Further longitudinal studies are needed to identify the influence of training load parameters on inflammatory markers in runners of different levels and training background.

## Introduction

Running is an important natural ability of our species that has contributed to our survival and body adaptations (Bramble and Lieberman, 2004). In the Paleolithic Era, survival was dependent on hunting and gathering, and therefore it has been suggested that the ancient physical activity pattern included mostly prolonged, low-intensity physical activities, including endurance running, interspersed with high-intensity bursts of activity (O'Keefe et al., 2010; Boullosa et al., 2013). Nowadays, endurance running is probably the most popular sport worlwide and it is practiced for recreational, health and competitive purposes (Chiampas and Goyal, 2015).

There is a close link between endurance running and the activity of the immune system. The importance of this relationship has led to important investigations over the last decades. Previously, Peters and Bateman (1983) identified an increased prevalence of upper respiratory tract infection (URTI) in 150 runners following a 56.0 km ultramarathon. Subsequently, specialized literature has suggested that even highly trained individuals, when subjected to frequent strenuous exercise, could develop a pro-inflammatory condition that favors the onset of a number of health problems, including damage to myocardial cells and connective tissues, overload of the atria and right ventricle, coronary artery disease (CAD), and coronary artery calcification among others (Peters and Bateman, 1983; Febbraio and Pedersen, 2005; Petersen and Pedersen, 2005; Zaldivar et al., 2006; Mohlenkamp et al., 2007; Hubble et al., 2009; Nieman, 2009; Meeusen et al., 2013; O'Keefe and Lavie, 2013; Taylor et al., 2014). However, little is known about whether the development of these chronic pathologies is the result of an excess of training volume, intensity, or both, associated with an insufficient recovery, which often promotes an increased susceptibility to infections and subsequent reduction in performance (Smith, 2004; Zaldivar et al., 2006; Hubble et al., 2009). Furthermore, systematic and non-systematic inflammation after running might be related with functional overreaching (Steinacker et al., 2004). In contrast, it has been suggested that periodised training with adequate recovery may be associated with positive adaptations including an adequate balance between pro-inflammatory and anti-inflammatory responses (Febbraio and Pedersen, 2005; Petersen and Pedersen, 2005; Zaldivar et al., 2006).

A growing body of evidence highlights the importance of studying inflammation promoted by endurance running as a factor which is linked to the physiopathology of a number of cardiovascular diseases (Mohlenkamp et al., 2007). It has also been suggested a link between myocardial damage and small thrombotic or even atherosclerotic emboli following a marathon, or after a quick session of exercise, accompanied by a transient monocytosis (about 2 h) (Walsh et al., 2011). The tissue factor is known as the key initiator of coagulation, and is highly dependent on vascular injury and mediators of inflammation such as tumor necrosis factor alpha (TNF-α), which has been reported to increase during and predominantly after marathon running (O'Brien, 2012; Gill et al., 2015b).

Contrary to other endurance sports, eccentric muscle contractions play a key role in running exercises, leading up to the occurrence of different levels of damage in muscle, connective and bone tissues (Suzuki et al., 2003; Jarvinen et al., 2013). The repair of these tissues involves the presence of inflammatory cells into the damaged site, which stimulates the release of pro-inflammatory cytokines such as TNF-α and interleukin-1 beta (IL-1β), thus triggering inflammation (Nieman et al., 1989, 1990). However, little is known about the impact of this chronic cycle tissue damage and repair in runners.

On the other hand, it is also important to emphasize that signaling promoted by repeated muscle contractions as in running, stimulates the production of anti-inflammatory mediators by myocytes, especially interleukin-6 (IL-6), which acts as an inhibitor of pro-inflammatory cytokines such as TNF-α by stimulating the production of its soluble receptor antagonists (Pedersen, 2013). In addition, IL-6 also stimulates the production of interleukin-10 (IL-10) and interleukin-1 receptor antagonist (IL-1ra), generating an anti-inflammatory environment which may counterbalance the pro-inflammatory responses associated to repetitive eccentric actions (Pedersen and Febbraio, 2012).

Despite the growing body of evidence regarding the effects of endurance running on inflammation, the link between transient acute responses and chronic adaptations needs to be addressed (Gleeson, 2007). This information would be important to shed light on the possible role of the inflammatory *milieu* in the pathophysiology of a number of diseases, especially the cardiovascular ones. Thus, the aim of this systematic review was to investigate the effects of different doses (i.e., training and competitive loads) of endurance running on the acute and chronic inflammatory responses, and the immune effects of this practice on runners of different levels and training backgrounds.

## Methods

### Search strategy

A systematic review was conducted and the recommendations from the Preferred Reporting Itens for Systematic Review and Meta-Analyses (PRISMA) were considered (Liberati et al., 2009). The search strategies were reported to ensure the integrity of the results and allow the updating using the same methods to bring emerging evidence into the review. The Boolean and proximity operators were used and the search strategy was correctly adapted for each database used (Table 1) (Sampson et al., 2008, 2009). Studies were identified by searching the following electronic databases: PubMED/MEDLINE (via National Library of Medicine) (2000–2017) and Science Direct (Elsevier) (2000–2017). The last search was conducted in February 2017.

**Table 1:** Search strategies.

**Database**	**Search strategy**	**Hits**	**No. (%) of trials finally selected**
PubMED/MEDLINE—via national library of medicine	1. Inflammation AND aerobic exercise AND runners;	128	50
	2. Cytokines AND runn^*^ AND (marathon runners or novice runners);	64	
		Total: 192	
Science Direct (Elsevier)	1. “marathon runners” OR “novice runners” AND “cytokines”;	94	4
		Total: 94	
Other sources (reference lists of the papers that fulfilled the inclusion criteria were analyzed for the identification of additional studies)		Total: 19	6

* *Truncation or wildcard*.

Once the abstracts were reviewed, the complete versions of the papers that met the criteria were obtained. In addition, the reference lists of the papers that fulfilled the inclusion criteria were analyzed for identification of additional studies. The exclusion of studies with irrelevant content and duplicates was carried out after the title, abstract and full-text were read.

### Definition of terms

An “athlete” was defined according to the Medical Subject Headings (MeSH) and was considered to be an individual who has developed skills, physical fitness and strength, or who has participated in sports running (MeSH, 2015). We have considered the definition proposed by Stirling and Kerr (2006) that defines a “recreational athlete” as being an individual who plays on a sports team at an amateur level, works out 1–4 times a week, does not train and compete nationally or internationally, and does not train for the same activity for more than 8 h per week. Novice runners were those individuals who had not been running on a regular basis in the previous 12 months (10 km total in all training sessions in the previous 12 months), and recreational runners were considered as individuals running a mean of 24.94 km/week (Videbaek et al., 2015).

The following thesaurus terms registered in the database from MeSH were also used: “running,” “aerobic exercise,” “inflammation,” and “cytokines.” These terms were associated with the free terms “recreational runners,” “novice runners,” “marathon runners,” and “ultramarathon.”

### Inclusion and exclusion criteria

The inclusion criteria were as follows: randomized controlled trials (RCTs) and non-randomized controlled trials (NRCTs); studies investigating the acute and chronic effects of running on markers of inflammation in marathon runners, recreational runners and novice runners; the terms runners, marathon runners, recreational runners and novice runners should be cited in the paper; only healthy participants; only full-text article citations with no restriction on languages; with individuals aged over 19 as the World Health Organisation (WHO) defines adolescence as the period in human growth and development that occurs between childhood and adulthood, from ages 10–19 (WHO, 2015)^1^. Meeting abstracts, unpublished data, observational studies, review articles, studies using walking and jogging as independent variables, and studies on the effects of any kind of supplements on running, diet restrictions, use of devices (e.g., equipment, compression garments), comparisons between running and other sports, and effects of environmental conditions (ex. dry and hot) were excluded.

### Outcome measures

The outcome measures assessed for acute and chronic effects of marathon and recreational running were interleukin (IL): IL-6, IL-10, IL-8, IL-1ra, IL-1β, IL-2 and IL-12, TNF-α, C reactive-protein (CRP), interferon-gamma (IFN-γ), soluble receptors, and transformation growth factor-beta (TGF-β). These mediators were chosen after an initial analysis and review of the literature. They were identified as the main outcomes in studies published with marathon runners, recreational runners and novice runners (Nieman et al., 2005; Santos et al., 2007; Scott et al., 2011; Abbasi et al., 2013; Jee et al., 2013; Shin and Lee, 2013).

### Quality assessment

The quality and assessment of all eligible articles was evaluated using a modified version of the Downs and Black checklist (Downs and Black, 1998). Disagreements between authors were discussed and subsequently solved. This modified version consists of 27 objective questions (Downs and Black, 1998).

## Results

### Research strategy

**Figure 1:**
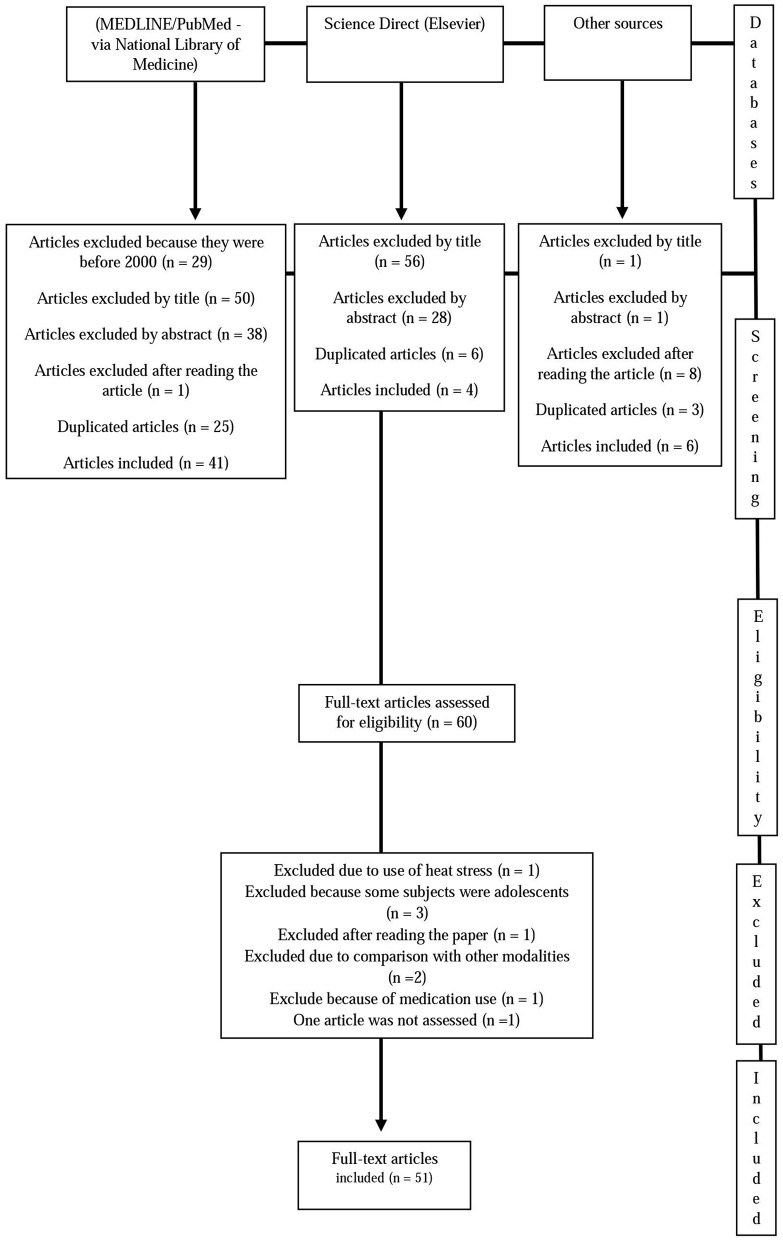
Summary of search results.

Results of the research strategy are presented in Figure 1. Initially, 60 studies were selected, with 51 studies being finally included according to the inclusion/exclusion criteria. Nine studies were excluded as follows: one study was excluded due to the use of heat stress, three because the subjects were adolescents, one following the reading of the full paper, two because of comparison with other sporting activities, and one because of medication use; in addition, one paper was not available in full-text version (Saravia et al., 2010). A total of 49 studies verified the acute effect of running on inflammation and two studies focused on the chronic effects.

### Methodological quality assessment

Quality assessment of the studies according to the modified Downs and Black scale is summarized in Table 2. One important finding was that characteristics of the patients' included were not cleary described in 32 studies. Important adverse events and description of patients' characteristics lost to follow-up was not reported in 34 and 32 studies, respectively. None of the studies were randomized controlled trials and power was provided in 4 studies (Table 2).

**Table 2:** Methodological quality assessment scores of the included studies.

**Study**	**Questions**																										
	**Reporting**										**External validity**			**Internal validity (bias)**							**Internal validity—confounding (selection bias)**						**Power**
	**1**	**2**	**3**	**4**	**5**	**6**	**7**	**8**	**9**	**10**	**11**	**12**	**13**	**14**	**15**	**16**	**17**	**18**	**19**	**20**	**21**	**22**	**23**	**24**	**25**	**26**	**27**
Grabs et al., 2015	1	1	1	1	1	1	1	0	0	1	0^*^	0^*^	1	0^*^	0^*^	0	1	1	0^*^	1	1	1	0	0	0	0	0
Fallon et al., 2001	1	1	1	1	1	1	1	1	0	1	0^*^	0^*^	1	0^*^	0^*^	0	1	1	0^*^	1	0	1	0	0	0	0	0
Kim et al., 2015	1	1	1	1	1	1	1	0	0	0	0^*^	0^*^	1	0^*^	0^*^	0	1	1	0^*^	1	0	0	0	0	0	0	1
Gill et al., 2015b	1	1	0	1	1	1	1	0	0	1	0^*^	0^*^	1	0^*^	0^*^	0	1	1	0^*^	1	1	1	0	0	0	0	0
Mattusch et al., 2000	1	1	0	1	1	1	1	0	0	0	0^*^	0^*^	1	0^*^	0^*^	0	1	1	0^*^	1	0	1	0	0	0	0	0
Neidhart et al., 2000	1	1	0	1	1	1	1	0	0	1	0^*^	0^*^	1	0^*^	0^*^	0	1	1	0^*^	1	0	0	0	0	0	0	0
Vaisberg et al., 2013	1	1	0	1	1	1	1	0	0	1	0^*^	0^*^	1	0^*^	0^*^	0	1	1	0^*^	1	1	0	0	0	0	0	0
Niess et al., 2000	1	1	0	1	1	1	1	0	0	0	0^*^	0^*^	1	0^*^	0^*^	0	1	1	0^*^	1	0	0	0	0	0	0	0
Kasprowicz et al., 2013	1	1	0	0	1	1	1	0	0	0	0^*^	0^*^	1	0^*^	0^*^	0	1	1	0^*^	1	0	0	0	0	0	0	0
Saugy et al., 2013	1	1	0	1	1	1	1	0	1	1	0^*^	0^*^	1	0^*^	0^*^	0	1	1	0^*^	1	0	1	0	0	0	1	0
Jee et al., 2013	1	1	1	1	1	1	1	0	1	0	0^*^	0^*^	1	0^*^	0^*^	0	1	1	0^*^	1	0	0	0	0	0	1	1
Karstoft et al., 2013	1	1	0	1	1	1	1	1	1	0	0^*^	0^*^	1	0^*^	0^*^	0	1	1	0^*^	1	0	0	0	0	0	1	0
Wilhelm et al., 2014	1	1	1	1	1	1	1	1	1	1	0^*^	0^*^	1	0^*^	0^*^	0	1	1	0^*^	1	1	1	0	0	0	1	0
Reihmane et al., 2013	1	1	0	1	1	1	1	1	0	0	0^*^	0^*^	1	0^*^	0^*^	0	1	1	0^*^	1	0	0	0	0	0	0	0
Millet et al., 2011	1	1	0	1	1	1	1	0	1	1	0^*^	0^*^	1	0^*^	0^*^	0	1	1	0^*^	1	0	0	0	0	0	0	0
Auersperger et al., 2012	1	1	1	1	1	1	1	1	1	1	0^*^	0^*^	1	0^*^	0^*^	0	1	1	0^*^	1	0	0	0	0	1	1	0
Bernecker et al., 2013	1	1	1	1	1	1	1	1	1	1	0^*^	0^*^	1	0^*^	0^*^	0	1	1	0^*^	1	1	1	0	0	1	1	0
Chimenti et al., 2009	1	1	0	1	1	1	1	1	0	0	0^*^	0^*^	1	0^*^	0^*^	0	1	1	0^*^	1	1	1	0	0	0	0	0
Papassotiriou et al., 2008	1	1	0	1	1	1	1	0	0	0	0^*^	0^*^	1	0^*^	0^*^	0	1	1	0^*^	1	1	1	0	0	0	0	0
Kim et al., 2007	1	1	0	1	1	1	1	0	0	1	0^*^	0^*^	1	0^*^	0^*^	0	1	1	0^*^	1	1	0	0	0	0	0	0
Peters et al., 2004	1	1	1	1	1	1	1	1	0	0	0^*^	0^*^	1	0^*^	0^*^	0	1	1	0^*^	1	1	1	0	0	0	0	0
Suzuki et al., 2003	1	1	0	1	1	1	1	0	0	1	0^*^	0^*^	1	0^*^	0^*^	0	1	1	0^*^	1	1	1	0	0	0	0	0
Bachi et al., 2015	1	1	0	1	1	1	1	0	0	1	0^*^	0^*^	1	0^*^	0^*^	0	1	1	0^*^	1	1	1	0	0	0	0	0
Kłapcinska et al., 2013	1	1	0	1	1	1	1	1	0	1	0^*^	0^*^	1	0^*^	0^*^	0	1	1	0^*^	1	1	0	0	0	0	0	0
Rehm et al., 2013	1	1	1	1	1	1	1	0	1	1	0^*^	0^*^	1	0^*^	0^*^	0	1	1	0^*^	1	1	1	0	0	0	1	0
Fehrenbach et al., 2000	1	1	0	1	1	1	1	1	1	0	0^*^	0^*^	1	0^*^	0^*^	0	1	1	0^*^	1	0	0	0	0	0	1	0
Schobersberger et al., 2000	1	1	0	1	1	1	1	0	1	0	0^*^	0^*^	1	0^*^	0^*^	0	1	1	0^*^	1	1	0	0	0	0	1	0
Suzuki et al., 2000	1	1	1	1	1	1	1	0	1	0	0^*^	0^*^	1	0^*^	0^*^	0	1	1	0^*^	1	1	1	0	0	0	1	0
Vaisberg et al., 2012	1	1	0	1	1	1	1	0	0	1	0^*^	0^*^	1	0^*^	0^*^	0	1	1	0^*^	1	0	0	0	0	0	0	0
Tomaszewski et al., 2003	1	1	1	1	1	1	1	0	0	1	0^*^	0^*^	1	0^*^	0^*^	0	1	1	0^*^	1	1	1	0	0	1	0	0
Bonsignore et al., 2002	1	1	0	1	1	1	1	0	0	1	0^*^	0^*^	1	0^*^	0^*^	0	1	1	0^*^	1	1	1	0	0	0	0	0
Nickel et al., 2012	1	1	1	1	1	1	1	0	0	0	0^*^	0^*^	1	0^*^	0^*^	0	1	1	0^*^	1	0	0	0	0	0	0	0
Waśkiewicz et al., 2012	1	1	0	1	1	1	1	0	0	0	0^*^	0^*^	1	0^*^	0^*^	0	1	1	0^*^	1	0	0	0	0	0	0	0
Chimenti et al., 2010	1	1	0	1	1	1	1	0	0	0	0^*^	0^*^	1	0^*^	0^*^	0	1	1	0^*^	1	0	0	0	0	0	0	0
Ng et al., 2008	1	1	0	1	1	1	1	1	1	0	0^*^	0^*^	1	0^*^	0^*^	0	1	1	0^*^	1	1	0	0	0	0	1	0
Siegel et al., 2007	1	1	0	1	0	1	1	0	0	1	0^*^	0^*^	1	0^*^	0^*^	0	1	1	0^*^	1	1	1	0	0	0	0	0
Shin and Lee, 2013	1	2	1	1	1	1	1	1	1	1	0^*^	0^*^	1	0^*^	0^*^	0	1	1	0^*^	1	1	0	0	0	0	1	0
Jee and Jin, 2012	1	1	1	1	1	1	1	0	1	0	0^*^	0^*^	1	0^*^	0^*^	0	1	1	0^*^	1	1	0	0	0	0	1	0
Santos et al., 2013	1	1	0	1	1	1	1	0	0	1	0^*^	0^*^	1	0^*^	0^*^	0	1	1	0^*^	1	1	1	0	0	0	0	0
Hewing et al., 2015	1	1	1	1	1	1	1	1	0	1	0^*^	0^*^	1	0^*^	0^*^	0	1	1	0^*^	1	1	1	0	0	1	0	0
Nieman et al., 2003	1	1	1	1	1	1	1	1	1	1	0^*^	0^*^	1	0^*^	0^*^	0	1	1	0^*^	1	1	0	0	0	0	1	0
Uchakin et al., 2003	1	1	0	1	1	1	1	0	0	1	0^*^	0^*^	1	0^*^	0^*^	0	1	1	0^*^	1	1	1	0	0	0	0	1
Mrakic-Sposta et al., 2015	1	1	0	1	1	1	1	0	1	0	0^*^	0^*^	1	0^*^	0^*^	0	0	1	0^*^	1	1	0	0	0	0	1	1
Stuempfle et al., 2016	1	1	1	1	1	1	1	1	1	1	0^*^	0^*^	1	0^*^	0^*^	0	1	1	0^*^	1	1	1	0	0	0	1	0
Nieman et al., 2016	1	1	0	1	1	1	1	0	0	1	0^*^	0^*^	1	0^*^	0^*^	0	1	1	0^*^	1	1	0	0	0	0	0	0
Arakawa et al., 2016	1	1	0	1	1	1	1	0	0	0	0^*^	0^*^	1	0^*^	0^*^	0	1	1	0^*^	1	1	0	0	0	0	0	0
Mohamed et al., 2016	1	1	0	1	1	1	1	0	0	0	0^*^	0^*^	1	0^*^	0^*^	0	1	1	0^*^	1	1	0	0	0	0	0	0
Cairns and Hew-Butler, 2015	1	1	1	1	1	1	1	1	1	1	0^*^	0^*^	1	0^*^	0^*^	0	1	1	0^*^	1	1	0	0	0	0	1	0
Gill et al., 2015a	1	1	0	1	0	1	1	1	0	1	0^*^	0^*^	1	0^*^	0^*^	0	1	1	0^*^	1	1	1	0	0	0	0	0
Krzeminski et al., 2016	1	1	0	1	1	1	1	0	0	0	0^*^	0^*^	1	0^*^	0^*^	0	1	1	0^*^	1	0	0	0	0	0	0	0
Nielsen et al., 2016	1	1	1	1	0	1	1	0	0	1	0^*^	0^*^	1	0^*^	0^*^	0	1	1	0^*^	1	1	0	0	0	0	0	0

*1 = yes and 0 = no for questions 1, 2, 3, 4, 6, 7, 8, 9, and 10*.

*For question 5: 2 = yes, 1 = partially, and 0 = no*.

For other questions 1 = yes, 0 = no, and 0* = unable to determine.

### Characteristics of the studies and summary of outcome measures

An overview of the studies' characteristics is provided in Table 3 with sample size, age, sex, and exercise protocols. A summary of outcome measures in selected studies is presented on Table 4.

**Table 3:** Characteristics of the studies.

**Citation**	**Age (years)**	**Exercise group (*N*) sex**	**Total subjects (*N*) male/female**	**Control group (*N*) sex**	**Level of runners**	**Method (distance)**	**Intervention (effects)**	
							**Acute**	**Chronic**
Grabs et al., 2015	45.0 ± 8.0	20	20 (♂)	–	ATL	Marathon	■	
Fallon et al., 2001	47.0 ± 7.0	8	8 (7♂-1♀)	–	ATL	Ultra-Marathon: 6 days	■	
Kim et al., 2015	50.8 ± 8.2	40	40 (♂)	–	LNR	Marathon	■	
Gill et al., 2015b	41.0 ± 8.0 49.0 ± 4.0	19 (13♂-6♀)	31 (18♂-13♀)	12 (5♂-7♀)	LNR	Ultra-marathon: 230 km Five stage (37, 48, 38, 69, 39 km)	■	
Mattusch et al., 2000	EG: 25–40 CG: 31–52	14	25 (♂)	11	REC	Training		■
Neidhart et al., 2000	EG: 25–34 CG: 24–35	8	24 sex (NR)	16	REC	Marathon	■	
Vaisberg et al., 2013	41.4 ± 9.4	Asymptomatic: 15 Symptomatic: 7	22 (♂)		ATL	Marathon	■	
Niess et al., 2000	EG: 32.3 ± 3.3 CG: 25.0 ± 2.2	$\frac{1}{2}$ Marathon Group: 10	18 (♂)	8	NOV	Half-marathon and treadmill	■	
Kasprowicz et al., 2013	44.5 ± 13.5	6	6 (♂)	–	ATL	Ultra-marathon: 100 km	■	
Saugy et al., 2013	45.4 ± 10.3 CG: 29.3 ± 8.1	25	33 (♂)	8	ATL	Ultra-marathon: 330 km (Mountain)	■	
Jee et al., 2013	EG: 49.75 ± 5.65 CG: 46.75 ± 5.44	8	16 (♂)	8	ATL	Ultra-marathon: 308 km	■	
Karstoft et al., 2013	44 ± 2	8	7 (♂) 1 (♀)	–	ATL	Marathon	■	
Wilhelm et al., 2014	34.9 ± 4.2	11	11 (♂)	–	ATL	Marathon (Mountain)	■	
Reihmane et al., 2013	Half-Marathon: 26 ± 5 Marathon: 27 ± 5	22 (♂) 18 (♂)	40 (♂)	–	REC	Half-marathon Marathon	■	
Millet et al., 2011	40.2	22	22 (♂)	–	ATL	Ultra-marathon 166 km	■	
Auersperger et al., 2012	Interval Group: 32.9 ± 5.7 Continuous Group: 31.6 ± 4.8	10 8	18 (♀)		REC	Chronic Training		■
Bernecker et al., 2013	43 (33–53)	12	12 (♂)		REC	Marathon	■	
Chimenti et al., 2009	40.3 ± 3.8	9 (♂)	9 (♂)		REC	Half-marathon, fall (21 km), winter (12 km) and summer (10 km)	■	
Papassotiriou et al., 2008	42.8 ± 1.4	15	15 (♂)		ATL	Ultra-marathon 246 km	■	
Kim et al., 2007	45.7 ± 5.1	54	54 (♂)		ATL	Ultra-marathon 200 km	■	
Peters et al., 2004	Fast group: 35.4 ± 1.84 Slow group: 41.4 ± 2.77	9 10	30 (♂)	–	ATL	Ultra-marathon 90 km	■	
Suzuki et al., 2003	31.7 ± 5.0	10	10 (♂)	–	ATL	Marathon	■	
Bachi et al., 2015	Sedentary group: 35.5 ± 7 Marathon runners: 35.7 ± 9	20	40 (♂)	20	REC	Marathon	■	
Kłapcinska et al., 2013	45.4 ± 9.2	7	7 (♂)		ATL	Ultra-marathon 48 h	■	
Rehm et al., 2013	40.95 ± 9.38	19	19 (14♂-5♀)		REC	Marathon	■	
Fehrenbach et al., 2000	32.3 ± 9.3	12	24 (♂)	12	REC	Half-marathon	■	
Schobersberger et al., 2000	36.3 (22–50)	13	13 (♂)	–	ATL	Ultra-marathon 67 km	■	
Suzuki et al., 2000	21–39	16	16 (♂)	–	ATL	Marathon	■	
Vaisberg et al., 2012	Sedentary Group: 37.5 ± 4 Athletes Group: 38 ± 7	14	42 (♂)	28	REC	Marathon	■	
Tomaszewski et al., 2003	Lean, BMI < 25 kg/m2 Marathon runners: 43.1 ± 8.4 Control: 42.5 ± 10.4 Non-lean, BMI > 25 kg/m2 Marathon runners: 45.6 ± 12.3 Control: 43.1 ± 7.5	55 12	110 (♂)	30 13	ATL	Ultra-marathon	■	
Bonsignore et al., 2002	41.3 ± 13.4	Half-marathon: 8 Marathon: 8	25 (♂)	9	ATL	Half-marathon and Marathon	■	
Nickel et al., 2012	LE: 40 ± 7; LNE 40 ± 6; ONE 40 ± 6	LE: 16; LNE: 16	47 (♂)	15	ATL and REC	Marathon	■	
Waśkiewicz et al., 2012	43.0 ± 10.8	14	14 (♂)	–	ATL	Ultra-marathon 24 h	■	
Chimenti et al., 2010	NR	15	15 (♂)	–	ATL	Half-marathon	■	
Ng et al., 2008	25 (21–32)	30	30 (♂)	–	NRL	Half-marathon	■	
Siegel et al., 2007	49 ± 10	33	33 (♂)	–	NRL	Marathon	■	
Shin and Lee, 2013	52.8 ± 5.0	18	18 (♂)	–	ATL	Ultra-marathon 308 km	■	
Jee and Jin, 2012	49.5 (47–54)	24	24 (♂)	–	ATL	Ultra-marathon 308 km	■	
Santos et al., 2013	Athletes 35.2 ± 3.6 Non-athletes 31.6 ± 2.3	Athletes: 15	27 (♂)	Non-athletes: 12	ATL	Marathon	■	
Hewing et al., 2015	50.3 (22–72)	167	167 (78♂) and (89♀)	–	ATL	Marathon	■	
Nieman et al., 2003	46.9 (33–65)	31	31 (22♂) and (9♀)	–	ATL	Ultra-marathon 160 km	■	
Uchakin et al., 2003	WR: 37.8 ± 3.9 CT: 40.3 ± 7.7	WR: 8	15	CT: 7	ATL	Marathon	■	
Mrakic-Sposta et al., 2015	45.04 ± 8.75	46	46 (♂)	–	ATL	Mountain Ultra-Marathon 330 km	■	
Stuempfle et al., 2016	With nausea: 44.3 ± 10.5 Without nausea: 41.8 ± 9.1	20	(15♂-05♀)	–	ATL	Ultra-Marathon 161-km	■	
Nieman et al., 2016	22–45	20	(10♂-10♀)	–	ATL	1.5 h on treadmills at ~70% VO_2_max followed by 30 min of downhill running	■	
Arakawa et al., 2016	52.1 ± 12.1	25	25 (♂)	–	ATL	Ultra-Marathon	■	
Mohamed et al., 2016	SS: 23.9 ± 4.20 LDR: 22.70 ± 3.70 MDR: 21 ± 1.80	SS (*n* = 8) LDR (*n* = 9) MDR (*n* = 8)	24 (♂)	–	ATL	Incremental Event (VAMEVAL test) Supra-Maximal Exhausting Race (Limited-Time Test)	■	
Cairns and Hew-Butler, 2015	43.7 ± 9.8	Normonatremic: 5 Hyponatremic: 10	15 (12♂ 3♀)	–	ATL	100 km (103.7 km) and 100 miles (173.7 km)	■	
Gill et al., 2015a	40 ± 7	17	(14♂-03♀)	17 (04♂-08♀)	ATL	Ultra-Marathon 24-H	■	
Krzeminski et al., 2016	30 ± 1.0	09	09 (♂)	–	ATL	Ultra-Marathon 100 km	■	
Nielsen et al., 2016	40 (29–56)	Half-Marathon: (09♂-09♀) Marathon: (14♂)	32	–	REC	Half-Marathon/Marathon		

*♂, male; ♀, female; EG, exercise group; CG, control group; PBMCs, peripheral blood mononuclear cells; LE, lean elite group; LNE, non-elite group; ONE, obese non-elite group; BMI, body mass index; WR, White Rock marathon; CT, CowTown marathon; SS, sedentary subjects; LDR, long-distance runners; MDR, Middle-distance runners; ALT, Athlete; REC, Recreational; NOV, Novice; Not reportable level (No reported details by authors), NRL; LNR, Level not reported*.

**Table 4:** Summary of outcome measures.

**Citation**	**Outcomes**	**Exercise Pre [mean (SD)] or median (IQR)**	**Post [mean (SD)] or median (IQR)**	**Control Pre [mean (SD)] or median (IQR)**	**Post [mean (SD)] or median (IQR)**	***p* value^b^**
Grabs et al., 2015	IL-6 (mg/L)	2.0 (0.0)	33.1 (24.1–37.00)^a^			
	hs-CRP (mg/L)	0.83 (0.57–1.18)	9.13 (6.48–13.63)^a^			
Fallon et al., 2001	CRP (mg/L)	0.19 (0.14)	1.84 (0.88)^a^			
Kim et al., 2015	hs-CRP (mg/L)	0.06 (0.07)	0.10 (0.09)^a^			
Gill et al., 2015b	CRP (mg/L)					
	Stage 1 Stage 2 Stage 3 Stage 4 Stage 5	1.1 (1.7) 7.4 (5.3)^†^ 10.0 (5.7)^†^ 9.2 (5.9)^†^ 8.8 (5.6)^†^	1.6 (2.4) 8.8 (5.4) 9.6 (5.9) 10.0 (6.7)^a^ 11.0 (6.4)^a^	1.4 (0.7) – 1.3 (0.8)^b^ – 1.3 (0.8)^b^		NS <0.05 <0.05
	IL-6 (pg/L) Stage 1 Stage 2 Stage 3 Stage 4 Stage 5	8.2 (4.5) 20.8 (18.5)^†^ 20.7 (16.8)^†^ 19.2 (14.1)^†^ 18.2 (11.6)^†^	27.9 (23.4)^a^ 20.7 (14.8) 25.3 (24.3)^a^ 21.7 (12.6)^a^ 23.4 (13.1)^a^	7.5 (2.5) – 5.5 (7.1)^b^ – 6.5 (5.7)^b^		NS <0.05 <0.05
	IL-1β (pg/L) Stage 1 Stage 2 Stage 3 Stage 4 Stage 5	0.6 (0.3) 1.1 (0.4)^†^ 1.2 (0.4)^†^ 1.1 (0.3)^†^ 1.2 (0.4)^†^	1.0 (0.3)^a^ 1.1 (0.4) 1.2 (0.4) 1.4 (0.4)^a^ 1.4 (0.4)^a^	0.7 (0.2) – 1.2 (0.2) – 1.3 (0.5)		NS NS NS
	TNF-α (pg/L) Stage 1 Stage 2 Stage 3 Stage 4 Stage 5	3.1 (2.9) 6.1 (4.5)^†^ 6.9 (4.4)^†^ 6.5 (4.2)^†^ 7.1 (3.8)^†^	6.3 (5.0)^a^ 6.6 (3.7) 6.1 (3.8)^a^ 8.1 (4.3)^a^ 8.3 (5.0)	1.3 (0.4) – 1.8 (0.7)^b^ – 2.3 (0.7)^b^		NS <0.05 <0.05
	IFN-γ (IU/ml) Stage 1 Stage 2 Stage 3 Stage 4 Stage 5	9.3 (5.5) 15.2 (6.8) 16.7 (6.7)^†^ 16.2 (7.2) 18.8 (10.0)^†^	12.9 (6.0)^a^ 16.9 (5.7) 15.2 (5.2) 19.9 (8.3)^a^ 22.7 (9.9)^a^	16.8 (5.5) – 14.3 (2.0) – 16.8 (5.1)		NS NS NS
	IL-10 (pg/ml) Stage 1 Stage 2 Stage 3 Stage 4 Stage 5	0.7 (0.6) 7.0 (10.8)^†^ 9.0 (10.2)^†^ 9.0 (12.2)^†^ 8.2 (11.2)^†^	7.9 (10.1)^a^ 7.9 (9.1) 8.0 (9.4) 9.3 (10.1) 10.9 (15.0)^a^	0.6 (0.1) – 1.4 (0.3)^b^ – 1.4 (0.7)^b^		NS <0.05 <0.05
	IL-1ra (pg/ml) Stage 1 Stage 2 Stage 3 Stage 4 Stage 5	22.9 (8.0) 39.8 (12.4)^†^ 45.5 (20.6)^†^ 37.9 (14.7)^†^ 47.1 (22.4)^†^	70.3 (28.1)^a^ 61.0 (39.8)^a^ 53.9 (19.0)^a^ 56.3 (30.4)^a^ 63.2 (28.1)^a^	23.4 (7.3) – 36.4 (9.2)^b^ – 33.1 (9.3)^b^		NS <0.05 <0.05
Mattusch et al., 2000	CRP (mg/L)	1.19 (1.63)	0.82 (0.94)	0.77 (2.18)	1.55 (9.17)	NS
Neidhart et al., 2000	CRP (mg/L) Before run (T0) After 31 km (T1) After 42 km (T2) 2 h after run (T3) 24 h after run (T4) 48 h after run (T5)	NR NR NR NR NR^b^^↑^^,^^c^^↑^ NR^b^^↑,^^c^^↑^		NR NR NR NR NR NR	NR NR NR NR NR NR	NS NS NS NS <0.05 <0.05
	IL-1β (ng/ml) Before run (T0) After 31 km (T1) After 42 km (T2) 2 h after run (T3) 24 h after run (T4) 48 h after run (T5)	NR NR NR NR NR NR		NR NR NR NR NR NR	NR NR NR NR NR NR	NS NS NS NS NS NS
	IL-1ra (ng/ml) Before run (T0) After 31 km (T1) After 42 km (T2) 2 h after run (T3) 24 h after run (T4) 48 h after run (T5))	95 260^b^^↑,^^c^^↑^ 485^b^^↑,^^c^^↑^ 1195^b^^↑,^^c^^↑^ NR NR		NR NR NR NR NR NR	NR NR NR NR NR NR	NS <0.05 <0.05 <0.05 NS NS
	IL-6 (ng/ml) Before run (T0) After 31 km (T1) After 42 km (T2) 2 h after run (T3) 24 h after run (T4) 48 h after run (T5)	1.8^b^^↓^ 8.7^c^^↑^ 9.8^c^^↑^ 5.3^c^^↑^ 2.2 2.1		NR NR NR NR NR NR	NR NR NR NR NR NR	<0.05 NS NS NS NS NS
	TNF-α (pg/ml) Before run (T0) After 31 km (T1) After 42 km (T2) 2 h after run (T3) 24 h after run (T4) 48 h after run (T5)	9.7 16.6^c^^↑^ 14.3^c^^↑^ 15.1^c^^↑^ 13.1^c^^↑^ 10.2^c^^↑^		NR NR NR NR NR NR	NR NR NR NR NR NR	NS NS NS NS NS NS
	sIL-6R (pg/ml) Before run (T0) After 31 km (T1) After 42 km (T2) 2 h after run (T3) 24 h after run (T4) 48 h after run (T5)	NR^b^^↑^ NR NR NR NR NR		NR NR NR NR NR NR	NR NR NR NR NR NR	<0.05 NS NS NS NS NS
	sTNFRII (pg/ml) Before run (T0) After 31 km (T1) After 42 km (T2) 2 h after run (T3) 24 h after run (T4) 48 h after run (T5)	3180 NR NR NR NR NR		NR NR NR NR NR NR	NR NR NR NR NR NR	NS NS NS NS NS NS
Vaisberg et al., 2013	Il-6 nasal cell extract (pg/mg) Baseline Immediately 72 h	Symptomatic 0.07 (0.11) 0.33 (0.17)^d^^↑^ 2.49 (2.35)^d^^↑^		Asymptomatic 0.22 (0.47) 0.49 (0.89)^d^^↑^ 0.94 (1.1)^d^^↑^		NS NS NS
	Il-6 serum (pg/ml) Baseline Immediately 72 h	8.2 (20.8) 40.9 (30.9)^d^^↑^ 10.6 (20.4)		14.9 (32.3) 39.2 (26.5)^d^^↑^ 16.9 (34.3)		NS NS NS
	IL-10 nasal cell extract (pg/mg)					
	Baseline Immediately 72 h	0.22 (0.30) 0.15 (0.15) 5.33 (8.00)^d^^,^^e^^↑^		0.28 (0.69) 1.0 (2.68) 10.26 (13.31)^b^^,^^d^^,^^e^^↑^		NS NS NS
	IL-10 serum (pg/ml) Baseline Immediately 72 h	0.76 (0.1)^b^ 57.2 (32.7)^d^^↑^ 1.1 (0.9)		17.4 (34.0) 30.7 (25.3)^d^^↑^ 4.5 (11.4)		<0.05 NS NS
Niess et al., 2000	IL-8 (pg/ml) Baseline 0 h 3 h 24 h 48 h TNF-α^**^	NR NR↑^d^^,^^b^ NR NR NR		NR NR NR NR NR		– <0.05 – – –
Kasprowicz et al., 2013	CRP (ng/ml) Baseline 25 km 50 km 75 km Post 14 h after	NR NR NR NR NR↑^d^^,^^f^ NR↑^d^^,^^f^^,^^g^				
	IL-6 (pg/ml) Baseline 25 km 50 km 75 km Post 14 h after	NR NR↑^d^ NR↑^d^^,^^f^ NR↑^d^^,^^f^ NR↑^d^^,^^f^^−^ NR↓^g^^,^^h^^,^^i^				
Saugy et al., 2013	CRP (mg/dl)	0.31 (0.32)	13.11 (7.51)^a^	1.05 (1.04)	0.65 (0.61)^b^	<0.05
Jee et al., 2013	CRP (mg/dl) Baseline 100 km 200 km 308 km	0.31 (0.21) 3.97 (4.58) 25.37 (18.24)^d^^,^^j^ NR		0.39 (0.61) 4.27 (5.75) 25.09 (14.54)^d^^,^^j^ 22.48 (12.90)^d^^,^^j^		NS NS NS NS
Karstoft et al., 2013	CRP (mg/dl)	1.0 (0.0)	6.0 (1.1)^a^	–	–	–
Wilhelm et al., 2014	TNF-α (pg/ml) Baseline Post race Follow-up 1 Follow-up 2	NR NR↑^d^ NR NR				
	IL-6 (pg/ml) Baseline Post race Follow-up 1 Follow-up 2	NR NR↑^d^ NR NR				
	hsCRP (mg/dl) Baseline Post race Follow-up 1 Follow-up 2	NR NR NR↑^d^ NR				
Reihmane et al., 2013	IL-6 (pg/ml)–Half-marathon			Marathon runners		
	Pre-race 15 min post-race 28 h post-race	NR NR↑^*^^b^ NR		NR NR NR		
	TNF-α (pg/ml)–Half-marathon					
	Pre-race 15 min post-race 28 h post-race	NR NR↑^*^^b^ NR		NR NR NR		
Saravia et al., 2010	**THE PAPER WAS NOT AVAILABLE**					
Millet et al., 2011	CRP (mg/dl) Pre Post 2 days after 5 days after 9 days after 16 days after	2.0 (0.0) 46.8 (24.8)^*^ 30.0 (19.7)^*^ 7.2 (3.7)^*^ 2.5 (1.2) 2.3 (0.6)				
Auersperger et al., 2012	IL-6 (pg/ml)^**^ Hs CRP (mg/dl) – Interval			Continuos		
	Baseline 3 weeks of training Recovery 1 3 weeks of training Recovery 2 Post	0.88 (1.40) 1.13 (1.45) 0.50 (0.66) 0.98 (0.88) 0.49 (0.59) 4.85 (12.54)		1.48 (1.04) 1.27 (0.99) 1.62 (2.92) 1.60 (1.52) 1.92 (2.99) 1.01 (1.28)		
Bernecker et al., 2013	IL-6 (ng/L)	2.06 (1.98–2.20)	31.93 (20.68–41.47)^a^			
	TNF-α (ng/L)	9.01 (7.16–10.26)	10.26 (9.33–12.31)^a^			
Chimenti et al., 2009	IL-8 (ng/ml)–Fall Baseline Race	NR NR				
	TNF-α (pg/ml)–Fall Baseline Race	NR NR				
	IL-8 (ng/ml)–Winter Baseline Race	NR NR				
	TNF-α (pg/ml)–Winter Baseline Race	NR NR				
	IL-8 (ng/ml)–Summer Baseline Race	NR NR				
	TNF-α (pg/ml)–Summer Baseline Race	NR NR				
Papassotiriou et al., 2008	CRP (mg/L) Baseline End of race 48 h post	0.8 (0.1) 93.0 (12.6)^d^ 70.6 (11.5)^d^^,^^k^				
	IL-6 (ng/L) Baseline End of race 48 h post	0.8 (0.1) 8376.0 (1819.8)^d^ 0.7 (0.1)^d^^,^^k^				
	TNF-α (ng/L) Baseline End of race 48 h post	3.9 (0.9) 4.0 (0.8)^d^ 3.7 (0.7)^d^^,^^k^				
Kim et al., 2007	Hs CRP (IU/L) Pre 100 km 200 km	2.0 (4.0) 6.0 (6.0)^*^ 46.0 (28.0)^*^^,^^j^				
	IL-6 (pg/ml) Pre 100 km 200 km	0.86 (0.17) 104.3 (45.5) 108.6 (28.4)^j^				
	TNF-α Pre 100 km 200 km	2.35 (1.56) 2.60 (1.43) 2.77 (1.82)				
Peters et al., 2004	CRP (mg/l)–Well trained	NR	NR↑^a^	Less trained NR	NR↑^a^	
Suzuki et al., 2003	TNF-α (pg/ml) Plasma Urine^**^	0.31 (0.44)	0.29 (0.38)			
	IL1-β (pg/ml) Plasma Urine	0.43 (0.27) 1.7 (3.7)	0.52 (0.23) 7.1 (5.1)^a^			
	IL-6 (pg/ml) Plasma Urine	1.27 (1.19) 2.86 (6.91)	101.40 (50.34)^a^ 23.60 (19.94)			
	IL-8 (pg/ml) Plasma Urine^**^	1.16 (0.70)	0.06 (6.95)^a^			
	IL-10 (pg/ml) Plasma Urine	8.0 (2.1) 19.3 (6.3)	32.8 (14.5)^a^ 22.8 (3.8)^a^			
Bachi et al., 2015	IL-8 (pg/ml) PBMCs Baseline Post 72 h post	NR↑^b^ NR NR↓^d^		NR NR NR		
	IL-8 (pg/ml) serum Baseline Post 72 h post	NR NR↑^d^ NR↓^l^		NR NR NR		
	IL-10 (pg/ml) PBMCs Baseline Post 72 h post	NR↑^b^ NR NR↓^d^		NR NR NR		
	IL-10 (pg/ml) serum Baseline Post 72 h post	NR NR↑^d^ NR↓^l^		NR NR NR		
Kłapcinska et al., 2013	CRP (mg/l) Baseline 12 h running 24 h running 48 h running 24 h post-race 48 h post-race	0.8 (0.8) 3.4 (17.7)^d^ 30.0 (8.9)^d^ 63.5 (31.5)^d^ 45.5 (37.8)^d^ 28.0 (38.2)^d^				
	IL-6 (pg/ml) Baseline 12 h running 24 h running 48 h running 24 h post-race 48 h post-race	0.64 (0.34) 35.86 (17.35)^d^ 33.25 (16.54)^d^ 23.20 (18.85)^d^ 7.39 (13.32)^d^ 2.19 (3.67)				
Rehm et al., 2013	sIFNγ (x1.000) pg/ml Baseline Pre-race Recovery	42.24 (26.75) 28.86 (23.16)^d^ 38.86 (28.76)				
	sIL4 pg/ml Baseline Pre-race Recovery	3.59 (5.08) 8.65 (10.71)^d^^,^^m^ 4.02 (6.06)				
	sIL10 pg/ml Baseline Pre-race Recovery	389.73 (254.22) 248.9 (191.8)^d^ 323.79 (240.5)				
Fehrenbach et al., 2000	IL-8 pg/ml Baseline 0 h 3 h 24 h	5.0 (6.5) 30.7 (5.3)^d^ 8.9 (11.3) 3.9 (6.3)				
	TNF-α pg/ml Baseline 0 h 3 h 24 h	0.3 (0.2) 1.2 (0.9) 0.6 (0.6) 0.3 (0.5)				
Schobersberger et al., 2000	IL-6 pg/ml Baseline 0 h 2 h Day1 Day3 Day5	0.0 (0–1.75) 60.0 (40.5–180.0)^d^ 65.0 (22.3–81.0)^d^ 0.0 (0.0–5.0) 0.0 (0.0–0.8) 0.0 (0.0–4.0)				
	IL1-ra pg/ml Baseline 0 h 2 h Day1 Day3 Day5	23.0 (18.0–33.5) 720.0 (370.0–42.01)^d^ 733.0 (443.0–41.63)^d^ 100.0 (43.0–221.0)^d^ 84.0 (25.0–246.0) 28.0 (17.0–215.0)				
	TNF-α pg/ml Baseline 0 h 2 h Day1 Day3 Day5	13.0 (10.3–15.0) 17.5 (15.0–30.0)^d^ 18.0 (14.3–24.8)^d^ 16.5 (12.8–28.0)^d^ 16.2 (11.0–17.5)^d^ 16.0 (12.0–20.0)^d^				
	sTNF-RI ng/ml Baseline 0 h 2 h Day1 Day3 Day5	2.4 (2.1–2.9) 5.7 (4.5–11.7)^d^ 6.2 (4.2–9.0)^d^ 3.9 (3.2–5.3)^d^ 3.5 (2.8–5.0)^d^ 2.8 (2.3–3.7)^d^				
	sTNF-RII ng/ml Baseline 0 h 2 h Day1 Day3 Day5	11.1 (7.2–13.9) 11.8 (10.4–22.4)^d^ 12.9 (10.6–21.3)^d^ 15.7 (10.4–19.7)^d^ 15.1 (9.8–19.9)^d^ 11.6 (8.1–17.7)^d^				
Suzuki et al., 2000	IL-1β pg/ml IL1-ra pg/ml IL-2 pg/ml IL-4 pg/ml IL-6 pg/ml IL-8 pg/ml IL-10 pg/ml IL-12 pg/ml^**^ TNF-α pg/ml^**^ IFN-α pg/ml^**^ IFN-γ pg/ml	1.8 (3.6) 59.0 (37.0) 73.0 (44.0) 3.5 (1.45) <1.1 (1.3) 22.0 (19) 13.9 (12.3) – – – 1.5 (0.8)	1.4 (2.1) 12629.0 (12360.0)^a^ 50.0 (49.0)^a^ 4.9 (4.1) 120.0 (79.0)^a^ 5.5 (25.0)^a^ 47.9 (23.1)^a^ – – – 1.4 (1.0)			
Vaisberg et al., 2012	IL-6 pg/ml Baseline Immediatly 72 h	NR NR^d^^,^^n^^↑^ NR		NR – –		NS NS NS
	TNF-α Baseline Immediatly 72 h	NR^b^^↑^ NR^d^^,^^n^^↑^ NR		NR – –		<0.05 NS NS
Tomaszewski et al., 2003 (64)	CRP mg/L	0.3 (0.2–0.7)	1.8 (1.0–3.4)^a^			
Bonsignore et al., 2002	TNF-α pg/ml–Half-marathon	TNF-α pg/ml–Marathon				
	Baseline End of race^d^^↑^ Post	NR NR NR		NR NR NR		
	IL-6 pg/ml–Half-marathon	IL-6 pg/ml–Marathon				
	Baseline End of race^d^^↑^ Post	NR NR^d^^,^^b^^↑^ NR		NR NR NR		NS NS NS
Tomaszewski et al., 2003	CRP mg/dl–Lean Elite Baseline Marathon^d^^↑^ 24 h^d^^↑^	CRP mg/dl–Lean Non-elite NR NR^d^^↑^ NR^d^^↑^		Obese non-elite NR NR^d^^↑^ NR^d^^↑^^b^^(in relation to Lean Elite)↑^		
	IL-6 pg/ml–Lean Elite Baseline Immediately post marathon^d^^↑^ 24 h^d^^↑,^^e^^↓^	IL-6 pg/ml–Lean non-elite NR^b^^(in relation Lean Elite)↑^ NR^d^^↑^ NR^d^^↑,^^e^^↓^		Obese non-elite NR^b^^(in relation to Lean Elite)↑^ NR^d^^↑^ NR^d^^↑,^^e^^↓^		
	TNF-α pg/ml–Lean Elite Baseline Immediately post marathon 24 h^d^^↑,^ ^e^^↑^	TNF-α pg/ml–Lean Non-elite NR NR NR^d^^↑,^^e^^↑^		Obese non-elite NR NR NR^d^^↑,^^e^^↑^		
	IL-10 pg/ml–Lean Elite Baseline ^b^^(in relation to obese non-elite)↓^ Immediately post marathon ^d^^↑^ 24 h^e^^↓^	IL-10 pg/ml–Lean Non-elite NR ^b^^(in relation to obese non-elite)↓^ NR^d^^↑^ NR^e^^↓^		Obese non-elite NR NR^d^^↑^ NR^e^^↓^		
Waśkiewicz et al., 2012	IL-6 mg/dL Baseline Immediately post Marathon Post 12 h Post 24 h	0.87 (0.68) 20.29 (7.77)^d^ 27.36 (7.67)^d^ 28.49 (11.99)^d^				
	hsCRP mg/dL Baseline Immediately post Marathon Post 12 h Post 24 h	1.7 (2.7) 1.7 (2.5) 8.7 (4.6)^d^ 39.2 (16.7)^d^				
Chimenti et al., 2010	IL-8 ng/ml–Octuber IL-8 ng/ml–May IL-8 ng/ml–November	NR NR NR	NR^a^ NR^a^ NR^a^			
Ng et al., 2008	IL-6 pg/ml IL-10 pg/ml IL-1ra TNF-α pg/ml IL1-β	9.2 (4.1)^a^ 6.4 (2.4)^a^ 154.1 (0.4) NR NR	15.2 (5.3) 9.6 (3.0) 189.8 (61.9) NR NR			
Siegel et al., 2007	IL-6 pg/mL Baseline 2 h post race 24 h post race	1.6 (0.45) 66.6 (11.9)^d^ 4.3 (0.6)				
	CRP ng/dL Baseline 2 h post race 24 h post race	0.10 (0.02) 0.10 (0.03) 2.3 (0.53)^d^				
Shin and Lee, 2013	CRP IU/L Baseline 100 km 200 km 308 km	NR NR^d^^↑^ NR^d^^↑^ NR^d^^↑^				
	IL-6 pg/ml Baseline 100 km 200 km 308 km	NR NR^d^^↑^ NR^d^^↑^ NR^d^^↑^				
	IL-10 pg/ml Baseline 100 km 200 km 308 km	NR NR^d^^↑^ NR^d^^↑^ NR^d^^↑^				
	IL-8 pg/ml Baseline 100 km 200 km 308 km	NR NR^d^^↑^ NR^d^^↑^ NR^d^^↑^				
Jee and Jin, 2012	hsCRP IU/L Baseline 100 km 200 km 308 km	0.40 (0.10) 5.06 (1.46)^d^ 25.56 (3.82)^d^^,^^i^ 21.87 (3.49)^d^^,^^i^				
	TNF-α pg/ml Baseline 100 km 200 km 308 km	3.68 (0.15) 4.00 (0.20) 3.37 (0.18)^i^ 4.50 (0.36)^d^^,^^o^				
Santos et al., 2013	IL-6 pg/ml IL1-ra pg/ml TNF-α pg/ml IL-8 pg/ml^**^ IL-10 pg/ml^**^ CRP UL	106.00 (38.5) 18.0 (12.0) 32.3 (13.3) – – 5.2 (0.5)	435.0 (145.5)^a^ 2708.0 (355.0)^a^ 32.4 (7.7) 40.4 (20.2) 32.0 (11.2) 5.3 (0.7)			
Hewing et al., 2015	CRP mg/dl Baseline Post 14 days lates	0.10 (0.05–0.21) 0.06 (0.04–0.12)^d^ 0.10 (0.06–0.18)				
Nieman et al., 2003	IL-10 pg/ml Baseline 90 km 160 km	4.65 (0.40) 39.7 (8.0)^d^ 49.0 (8.2)^d^				
	IL1-ra pg/ml Baseline 90 km 160 km	229.0 (14.0) 2330.0 (421.0)^d^ 1616.0 (255.0)^d^				
	IL-6 pg/ml Baseline 90 km 160 km	1.19 (0.15) 58.6 (4.6)^d^ 60.9 (9.4)^d^				
	IL-8 pg/ml Baseline 90 km 160 km	6.31 (1.09) 20.4 (2.1)^d^ 22.0 (2.4)^d^				
Uchakin et al., 2003	IL-2 (IU/ml) Baseline 0 h 1 h 24 h 48 h 5 days 8 days	6.0 (2.5) 2.0 (0.4)^d^ 1.6 (0.2)^d^ 6.6 (1.0) 5.0 (0.6) 8.4 (1.2)^d^ 5.7 (1.0)				
	INF-γ (IU/ml) Baseline 0 h 1 h 24 h 48 h 5 days 8 days	210.8 (26.6) 17.7 (3.5)^d^ 14.4 (3.3)^d^ 196.4 (15.8) 154.8 (20.0) 272.8 (23.9)^d^ 141.0 (18.1)				
	IL-10 (pg/ml) Baseline 0 h 1 h 24 h 48 h 5 days 8 days	445.3 (69.3) 310.0 (44.3) 463.7 (146.9) 262.3 (27.0) 288.2 (33.2) 441.8 (73.9) 355.0 (47.9)				
	TNF-α (pg/ml) Baseline 0 h 1 h 24 h 48 h 5 days 8 days	16937.0 (1800.8) 9594.0 (1421.5)^d^ 1394.0 (1522.8)^d^ 12859.0 (1585.0)^d^ 12899.0 (1720.8)^d^ 12276.0 (12276.7)^d^ 14043.0 (1231.2)				
	IL1-β (pg/ml) Baseline 0 h 1 h 24 h 48 h 5 days 8 days	4377.1 (664.5) 2937.1 (696.8) 3162.9 (617.1) 3520.0 (743.3) 2342.8 (359.6)^d^ 2388.6 (481.9)^d^ 2817.1 (243.6)				
	IL-6 (pg/ml) Baseline 0 h 1 h 24 h 48 h 5 days 8 days	16571.4 (2058.1) 13585.7 (3105.4) 16200.0 (1740.2) 6514.3 (985.0)^d^ 8414.3 (1470.9)^d^ 10642.8 (2291.1) 14271.4 (1331.8)				
Mrakic-Sposta et al., 2015	IL-6 (pg/ml) Plama IL-6 (pg/ml) Urine	1.29 ± 0.54 0.71 ± 0.17	66.42 ± 36.92^a^ 1.33 ± 0.56^a^			
Stuempfle et al., 2016	IL-6 (pg/ml) CRP (ng/ml)	Without Nausea 0.9 ± 0.4 323 ± 487	Without Nausea 105.7 ± 53.6^a^ 31,448 ± 13,149^a^	With Nausea 1.0 ± 0.7 1686 ± 2607	With Nausea 78.6 ± 62.5^a^ 46,361 ± 29,708^a^	NS NS
Nieman et al., 2016	IL-6 (pg/ml) Pre-run Post-run 1-h Post-run 24-h Post run IL-8 (pg/ml) Pre-run Post-run 1-h Post-run 24-h Post run IL-10 (pg/ml) Pre-run Post-run 1-h Post-run 24-h Post run IL-1ra (pg/ml) Pre-run Post-run 1-h Post-run 24-h Post run CRP (mg/l) Pre-run Post-run 1-h Post-run 24-h Post run	Male 3.17 ± 0.41 11.8 ± 2.23^*^ 9.03 ± 1.41^*^ 2.36 ± 0.31 9.99 ± 1.00 22.4 ± 2.9^*^ 18.1 ± 1.5^*^ 8.12 ± 0.50 2.50 ± 0.18 9.33 ± 1.77^*^ 10.5 ± 1.9^*^^b^ 2.51 ± 0.53 111 ± 10.8 216 ± 14.8 385 ± 83.8 111 ± 9.8 0.71 ± 0.15 0.67 ± 0.17 0.62 ± 0.16 2.56 ± 0.50^*^		Female 2.88 ± 0.91 7.46 ± 0.89 7.37 ± 1.56 2.99 ± 1.19 8.88 ± 0.68 16.1 ± 1.1 16.6 ± 2.1 9.17 ± 0.68 3.31 ± 0.64 6.20 ± 0.91 6.03 ± 0.81 3.51 ± 0.81 125 ± 18.4 215 ± 40.5 300 ± 80.4 117 ± 9.3 0.70 ± 0.19 0.71 ± 0.22 0.70 ± 0.19 2.02 ± 0.73		
Arakawa et al., 2016	IL-6 (pg/ml) Baseline Day 1 Day 2 Day 3 Day 5 Day 7 CRP (mg/dl) Baseline Day 1 Day 2 Day 3 Day 5 Day 7 TNF-α (pg/ml) Baseline Day 1 Day 2 Day 3 Day 5 Day 7	0.77 ± 0.26 26.52 ± 5.05^d^ 19.28 ± 1.99^d^ 3.35 ± 0.96 6.53 ± 4.16 1.40 ± 0.38 0.07 ± 0.03 0.07 ± 0.03 0.72 ± 0.14^d^ 1.45 ± 0.29^d^ 0.64 ± 0.18 0.57 ± 0.28 0.91 ± 0.06 0.95 ± 0.09 0.84 ± 0.06 0.95 ± 0.08 0.98 ± 0.07 1.15 ± 0.17				
Mohamed et al., 2016	IL-6 (pg/ml) VAMEVAL test Before After IL-6 (pg/ml) Limited-time test Before After TNF-α (pg/ml) VAMEVAL test Before After TNF-α (pg/ml) Limited-time test Before After	Sedentary Subjects NR NR^d^^↑^ NR NR^d^^,^^b^^↑^ NR NR^d^^↑^ NR NR^d^^↑^	Long-Distance Runners NR NR^d^^↑^ NR NR^d^^↑^ NR NR^d^^↑^ NR NR^d^^↑^	Middle-Distance Runners NR NR^d^^↑^ NR NR^d^^↑^ NR NR^d^^↑^ NR NR^d^^↑^		
Cairns and Hew-Butler, 2015	IL-6 (pg/ml) Before After	With hyponatremia 0.1 ± 0.2 10.6 ± 6.1^*^	Non-hyponatremia 0.5 ± 0.4 8.4 ± 2.8^*^			
Gill et al., 2015a	IL-6 (pg/ml) Before After IL-1β (pg/ml) Before After TNF-α (pg/ml) Before After IFN-γ (pg/ml) Before After IL-10 (pg/ml) Before After IL-8 (pg/ml) Before After	0.4 (0.3 to 0.5) 14.5 (9.3 to 19.7)^*^ 0.1 (0.0 to 0.3) 0.6 (0.1 to 1.1)^*^ 2.8 (2.5 to 3.2) 3.8. (3.5 to 4.2)^*^ 1.0 (0.6 to 1.4) 1.2 (0.3 to 2.2) 2.1 (1.3 to 2.9) 12.8 (7.3 to 18.2)^*^ 11.4 (9.4 to 13.4) 38.7 (26.3 to 51.1)^*^				
Krzeminski et al., 2016	TNF-α (pg/ml) Pre-race Post-race 90 min post-race IL-6 (pg/ml) Pre-race Post-race 90 min post-race IL-10 (pg/ml) Pre-race Post-race 90 min post-race IL-18 (pg/ml) Pre-race Post-race 90 min post-race IL-1β (pg/ml) Pre-race Post-race 90 min post-race	1.39 ± 0.09 1.63 ± 0.09^*^ 1.54 ± 0.09 0.54 ± 0.07 47.35 ± 8.48^*^ 37.67 ± 7.94^*^^l^ 0.31 ± 0.06 5.04 ± 1.34^*^ 1.24 ± 0.35^*^ 75.08 ± 9.46 96.74 ± 9.92^*^ 101.25 ± 9.28 0.76 ± 0.24 1.30 ± 0.27 0.70 ± 0.12				
Nielsen et al., 2016	IL-1β (pg/ml) Pre-race Post-race Il-6 (pg/ml) Pre-race Post-race IL-8 (pg/ml) Pre-race Post-race IL-10 (pg/ml) Pre-race Post-race TNF-α (pg/ml) Pre-race Post-race IFN-γ (pg/ml) Pre-race Post-race	Marathon NR NR NR NR^*^^↑^ NR NR^*^^↑^ NR NR^*^^↑^ NR NR NR NR		Half-Marathon NR NR NR NR^*^^↑^ NR NR^*^^↑^ NR NR NR NR NR NR		

NS, not significant; –, not compared or evaluated;

** , below the detectable plasma concentrations; NR, not reported; PBMCs, produced by peripheral blood mononuclear cells; IL-6, interleukin 6; CRP, C-reactive protein; IL-8, interleukin- eight; IL-2, interleukin two; IL-4, interleukin four; IL-10, interleukin tem; IL-12, interleukin 12; IFN-γ, interferon gama; TNF-α, turmor necroses factor alpha; IL1-ra, receptor antogonist of interleukin one; IL1-β, interleukin beta; sTNF-R, soluble receptor for turmor necroses factor alpha; sIL6-R, soluble receptor antagonista for interleukin six; 0 h, immediately post-race;

† Significant difference (p < 0.05) vs. pre-stage 1;

* Significant difference (p < 0.05) vs. pre;

a Significant difference (p < 0.05) between pre and post for the same group;

b Significant difference (p < 0.05) between groups;

c Significant difference (p < 0.05) vs. T0;

d Significant difference (p < 0.05) vs. baseline;

e Significant difference (p < 0.05) vs. immediately;

f Significant difference (p < 0.05) vs. 25 km;

g Significant difference (p < 0.05) vs. 50 km;

h Significant difference (p < 0.05) vs. 75 km;

i Significant difference (p < 0.05) vs. post;

j significant difference (p < 0.05) vs. 100 km;

k Significant difference (p < 0.05) vs. end of race;

l Significant difference (p < 0.05) vs. post;

m Significant difference (p < 0.05) vs. recovery;

n Significant difference (p < 0.05) vs. 72 h;

o *Significant difference (p < 0.05) vs. 200 km*.

The 51 studies included resulted in a total of 1,421 subjects, of whom 163 were female and 1,234 males; 24 subjects were not identified by sex in one study (Neidhart et al., 2000). All trials provided age ranges for the subjects and the mean age was 39.16 years.

The common protocols adopted included marathon in 17 studies, ultra-marathon in 22, half-marathon in three studies, different distance protocols (42.195, 21.1, 12, 10 km and treadmill) in seven studies, and chronic training only in two studies (see Table 3).

## Discussion

The aim of this systematic review was to analyze studies that verified the effects of different endurance running exercises on acute and chronic inflammatory responses in runners of different training background. The present systematic review allows an initial understanding of this issue. It seems that acute and chronic endurance running may affect anti- and pro-inflammatory markers. However, important differences between studies in terms of methods as well as in runners' charactersitics do not allow appropriate comparison or generalization of results.

### Inflammatory markers

The timing in data collection could be considered an important limiting factor for adequate comparisons, given the heterogeneity observed across the reported acute (i.e., 5–20 min) (Fallon et al., 2001; Kim et al., 2007; Vaisberg et al., 2013; Grabs et al., 2015) and delayed responses (24 h to 8 days) (Uchakin et al., 2003; Siegel et al., 2007; Hewing et al., 2015). This is not a simple issue given the different kinetics and biological availability of the molecules considered as IL-6 and CRP (Kasprowicz et al., 2013; Reihmane et al., 2013). Of note, some inflammatory markers (e.g., monocyte chemoattractant protein-1, granulocyte colony-stimulating factor) (Suzuki et al., 2003) have not been included in the current review. Further studies should elaborate appropriate study designs that consider both the appropriateness of the inflammatory markers selected as well as their kinetics.

### Runners' and training load characteristics

Another important confounding factor is the different experience of runners. Thus, 26 studies (4 studies with male and female participants, and 15 studies with only male participants) reported a range of 4–17.5 years of experience with competitions (e.g., marathon, and ultra-marathon experience) (Schobersberger et al., 2000; Fallon et al., 2001; Nieman et al., 2003, 2016; Suzuki et al., 2003; Tomaszewski et al., 2003; Kim et al., 2007, 2015; Millet et al., 2011; Jee and Jin, 2012; Vaisberg et al., 2012, 2013; Waśkiewicz et al., 2012; Bernecker et al., 2013; Jee et al., 2013; Karstoft et al., 2013; Kasprowicz et al., 2013; Kłapcinska et al., 2013; Saugy et al., 2013; Shin and Lee, 2013; Wilhelm et al., 2014; Grabs et al., 2015; Hewing et al., 2015; Mrakic-Sposta et al., 2015; Krzeminski et al., 2016; Mohamed et al., 2016). However, 25 studies did not report this information (Fehrenbach et al., 2000; Mattusch et al., 2000; Neidhart et al., 2000; Niess et al., 2000; Suzuki et al., 2000; Bonsignore et al., 2002; Uchakin et al., 2003; Peters et al., 2004; Siegel et al., 2007; Ng et al., 2008; Papassotiriou et al., 2008; Chimenti et al., 2009, 2010; Auersperger et al., 2012; Nickel et al., 2012; Rehm et al., 2013; Santos et al., 2013; Bachi et al., 2015; Gill et al., 2015b; Arakawa et al., 2016; Nielsen et al., 2016; Stuempfle et al., 2016; Vernillo et al., 2017). This is not a trivial issue given that training experience of runners could have a potentially additive effect to the influence of runners' age on inflammation that warrants further research. Besides, only 34 articles especified the training preparation (km/week that ranged between 21.4 and 161 and time that ranged between 3.9 and 10 h/week) of runners before races (Fehrenbach et al., 2000; Mattusch et al., 2000; Niess et al., 2000; Suzuki et al., 2000, 2003; Fallon et al., 2001; Bonsignore et al., 2002; Nieman et al., 2003, 2016; Tomaszewski et al., 2003; Uchakin et al., 2003; Peters et al., 2004; Chimenti et al., 2009; Millet et al., 2011; Auersperger et al., 2012; Jee and Jin, 2012; Nickel et al., 2012; Vaisberg et al., 2012, 2013; Waśkiewicz et al., 2012; Bernecker et al., 2013; Karstoft et al., 2013; Kłapcinska et al., 2013; Rehm et al., 2013; Reihmane et al., 2013; Santos et al., 2013; Shin and Lee, 2013; Wilhelm et al., 2014; Bachi et al., 2015; Grabs et al., 2015; Hewing et al., 2015; Mrakic-Sposta et al., 2015; Arakawa et al., 2016; Krzeminski et al., 2016). Furthermore, only two studies cited the control of intensity during training (Mattusch et al., 2000; Auersperger et al., 2012). This aspect would be important for a better understanding of the relationship between running and inflammation from a dose-response perspective. For instance, higher values for IL-6 after a limited-time test were observed in sedentary individuals when compared to long- and middle-distance runners, but with no differences between groups for TNF-α (Mohamed et al., 2016). Given the growing popularity of races that last various days, further studies are warranted to elucidate if chronic exposure to high volumes of endurance running are detrimental for health. From an evolutionary perspective, this is an interesting topic given that daily running volumes of modern hunter-gatherers are far below (e.g., ~10–15 km) (O'Keefe et al., 2010; Boullosa et al., 2013) the training and competitive volumes of runners competing in ultra-endurance events. In addition, another new aspect that must be raised in further studies is the imbalance between training phases and recovery not reported in the studies included in this systematic review. Thus, since functional overreaching might be related to inflammation (Steinacker et al., 2004), further studies should explore these relationship in conjunction with other biological markers of overreaching.

Another important characteristic for a better characterization of runners is their fitness level. For instance, maximum oxygen consumption (VO_2_max), which is the gold standard for aerobic evaluation, has been reported only in 14 articles (3 studies with participants of both sexes (Nieman et al., 2003, 2016; Ng et al., 2008; Chimenti et al., 2009; Jee et al., 2013), and 11 studies with males (Millet et al., 2011; Jee and Jin, 2012; Waśkiewicz et al., 2012; Kłapcinska et al., 2013; Shin and Lee, 2013; Wilhelm et al., 2014; Kim et al., 2015; Krzeminski et al., 2016; Mohamed et al., 2016). The participants of these studies could be considered recreational runners when classified by their actual VO_2_max (44–51 and 35–41 mL.kg-1.min-1, for males and females, respectively) (Martin and Coe, 2007). In contrast, no study included elite runners when classified by their actual VO_2_max (70–85 mL.kg-1.min-1 and 61–73 mL.kg-1.min-1 for males and females respectively) (Martin and Coe, 2007). Moreover, runners' classification in the current review has been challenging when using the selected criteria (see Table 3) (Stirling and Kerr, 2006; MeSH, 2015). Thus, further studies should provide all these informations for a better characterization of runners. As we did not perceive an influence of aerobic fitness on inflammatory markers, further studies should elaborate on this relationship while controlling other runners' characteristics as training experience. Additionally, the influence of other fitness components as muscle strength capacity should be assessed in further studies for verifying the potentially protective effect for muscle damage and therefore on inflammation.

Another important limitation for generalization of the results refers to the heterogeneity of running exercises (e.g., distance, intensity) used for evaluation of acute inflammatory responses. Furthermore, ambient characteristics (e.g., altitude, temperature) and race profile (e.g., uphill and downhill running) which have been suggested to influence muscle contraction and physiological responses (Vernillo et al., 2017), have not been always reported (Millet et al., 2011; Saugy et al., 2013). These aspects should be controlled in further studies for isolating the relative effect of every specific factor on inflammation.

### Body fatness and inflammation

Given the relationship between adipose tissue and inflammation (Pedersen and Febbraio, 2012), attention should be paid to overweight and obese runners. For instance, a higher level of CRP 24 h following a marathon has been observed in obese non-elite runners when compared to lean elite runners (Nickel et al., 2012). Furthermore, obese non-elite runners when compared to lean elite and lean non-elite runners demonstrated a higher level of IL-6 and a lower level of IL-10 serum levels at baseline (Nickel et al., 2012). However, all groups presented an increase for serum IL-10 and TNF-α, and a decrease for serum IL-6 levels, immediately post-marathon (Nickel et al., 2012). It must be considered that increments in IL-10 induced by exercise may be responsible for the elevation in IL1-ra which exerts an anti-inflammatory action by antagonizing IL-1 and IL-1β (Dinarello, 2000; Moldoveanu et al., 2001; Petersen and Pedersen, 2005; Pedersen, 2011). Nevertheless, it could be suggested that, in overweighted individuals, a higher pro-inflammatory status at baseline and post-marathon could be observed, with unknow consequences for health in the long term.

### Inflammation and cardiovascular health

One relevant issue refers to the link between inflammation and cardiovascular health. Interestingly, the exercise-induced increase of IL-6 after the marathon in 20 lean male runners was associated with a lower prevalence of arrhythmias during and after the marathon race (Grabs et al., 2015). When produced by muscle contraction, IL-6 stimulates the synthesis of other anti-inflammatory cytokines such as IL-1ra and IL-10, thus providing an inhibitory effect on pro-inflammatory cytokines such as IL-1β and TNF-α (Pedersen and Febbraio, 2012; Pedersen, 2013). However, CRP, a strong predictor of cardiovascular events, is an acute phase protein synthesized in the liver by the stimulation of IL-6 (Ridker et al., 2002). Chronic endurance training may decrease CRP values, especially when accompanied by a loss in fat mass, therefore promoting further reduction of risk for cardiovascular events (Fallon et al., 2001; Tomaszewski et al., 2003; Walsh et al., 2011; Grabs et al., 2015; Kim et al., 2015). Of note, CRP may be more susceptible to chronically decrease in individuals presenting higher baseline levels (Barnett et al., 2005). Therefore, caution should be taken when evaluating the anti- and pro-inflammatory effects of running in individuals with different characteristics regarding cardiovascular risk factors (e.g., body composition) (Moldoveanu et al., 2000; Petersen and Pedersen, 2005; Walsh et al., 2011).

### Studies' characteristics

Most studies included in this systematic review were acute interventions (49 studies). However acute changes in inflammatory markers might not be related with anti- and pro-inflammatory outcomes during chronic aerobic training interventions. For instance, there were divergent responses for CRP changes in chronic interventions (Mattusch et al., 2000; Auersperger et al., 2012). Thus, while Mattusch et al. (2000) observed a reduction in CRP levels, Auersperger et al. (2012) did not observe any change. Therefore, further studies must consider this important limitation, while providing training load charactersitics as volume, intensity, and frequency of training sessions. An important question to be answered refers to the minimal training load required for runners of different levels when preparing different competitive distances, while analyzing the impact of these factors on inflammatory markers. Additionally, there is a prevalence of male runners on literature therefore more studies with female runners are needed.

## Conclusion

In summary, our results revealed that acute and chronic endurance running may affect anti- and pro-inflammatory markes but methodological differences between studies do not allow comparisons or generalization of the results. Only two studies were chronic interventions. There are no studies with elite athletes. Thus, RCTs are urgently needed to identify the appropriate dose of endurance running (volume, intensity, and frequency) required to elicit improvements in inflammatory markers in runners of different levels and training background. External (e.g., ambient characteristics, race profile) and internal factors (e.g., fitness level, training experience) to runners should be considered in further studies for a better understanding of the relationship between running and the mediators of inflammation. The information provided in this systematic review would help practitioners for better designing further studies while providing reference values for a better understanding of inflammatory responses after different running events.

## Author contributions

Conception and design: EB, CC, ON, JP, and DB. Search: EB, DN, and FS. Eligibility and outcome measures: EB and DN. Quality assessment: DN and JP. Writing of the manuscript: EB, DN, JP, ON, CC, and DB. Revision and approval of the final manuscript version and interpretation of the results: EB, DN, JP, ON, CC, FS, and DB.

### Conflict of interest statement

The authors declare that the research was conducted in the absence of any commercial or financial relationships that could be construed as a potential conflict of interest.

## Footnotes

^1^World Health Organization; Available online at: http://www.who.int/maternal_child_adolescent/topics/adolescence/dev/en/ (Acessed November 1, 2015).
